# Supplementary material for: Middle-Aged and Older Adults’ Beliefs, Ratings, and Preferences for Receiving Multicomponent Lifestyle-Based Brain Health Interventions
Source: Brain Sci. 2026 Jan 2;16(1):69. doi: 10.3390/brainsci16010069 (PMC12839200; doi:10.3390/brainsci16010069)
Supplement: Supplementary file 1 [file brainsci-16-00069-s001.zip › brainsci-4049056-supplementary.pdf]

Brain Health Knowledge, Attitudes, and Preferences Questionnaire

Welcome to This Study

This is a research study to help us find out what people 40 years of age and older know about keeping their brains healthy as they get older. There are three sections:

1) Asking about how helpful you think brain health activities are (like exercise, brain training, and changing your diet) and whether you're doing it;
2) How concerned you are about your brain health and if you have family member who has dementia;
3) If you want to work on your brain health, what would be helpful to you (things like in-person or online classes, or apps for your phone).

Thanks for helping with this study. Because this is a research study, we need to ask your consent to be in it. The next page has important information that you may want to know about the study. After you read it, you can go on to the questions.

Participant Letter for Anonymous Surveys (v2023-08-31)
NSU Consent to be in a Research Study Entitled
Older Adults' Knowledge of and Interest in Brain Health Activities

Who is doing this research study?

The person doing this study is Raymond L Ownby, MD, PhD with the Department of Psychiatry and Behavioral Medicine.

Why are you asking me to be in this research study?

You are being asked to take part in this research study because you are 40 years of age or older.

Why is this research being done?

The purpose of this study is to find out what older adults know about how their lifestyles affect their brain health. It is also to find what they are doing for it and what they would do to improve it.

What will I be doing if I agree to be in this research study?

You will be taking a one-time, anonymous survey. The survey will take about 20 minutes to complete.

Are there possible risks and discomforts to me?

This research study involves minimal risk to you. To the best of our knowledge, the things you will be doing have no more risk of harm than you would have in everyday life.

What happens if I do not want to be in this research study?

You can decide not to be in this research and it will not be held against you. You can exit the survey at any time.

Will it cost me anything? Will I get paid for being in the study?

There is no cost for being in this study. Participation is voluntary. Payment will be based on how you have set up payments with SurveyMonkey.

How will you keep my information private?

Your responses are anonymous. Information we learn about you in this research study will be handled in a confidential manner, within the limits of the law. All data will be kept on SurveyMonkey’s encrypted servers. Then we will download it to our computers. To see the data, people have to enter a password. The data will be available to the researcher, the Institutional Review Board and other representatives of this institution. All confidential data will be kept securely on encrypted and password-protected computers accessed only by the researchers during data analyses. All data will be kept for 36 months from the end of the study. It will then be destroyed by erasing all data files from our computers.

Who can I talk to about the study?

If you have questions, you can contact Rosemary Davenport, APRN at 954-262-1804 or Dr. Ownby at 954-262-1481.

If you have questions about the study but want to talk to someone else who is not a part of the study, you can call the Nova Southeastern University Institutional Review Board (IRB) at (954) 262-5369 or toll free at 1-866-499-0790 or email at IRB@nova.edu.

Do you understand and do you want to be in the study?

If you have read the above information and voluntarily wish to be in this research study, please click on the next button below to begin the survey.

Brain Health Knowledge, Attitudes, and Preferences Questionnaire

In the first part of this questionnaire, we'd like for you to tell us what you think about things that may help people aged 40 and older stay brain healthy as they get older. These are things that people can do on their own that help reduce the chances that they will have problems with thinking or their memory when they get older. If you don't know or would prefer not to answer, just check one of the boxes on the right. We'd also like to know if you are doing this kind of thing now – for example, do you do exercise or follow a diet?

Here, "brain health" means keeping your brain working as well as you can.

Thanks for your help with this project!

Part 1: Rating Activities for Brain Health (there are 19 items in this section, with 2 answers for each, plus a space at the end for you to make suggestions). First we will ask some questions to help us understand who you, so that we can understand your responses in the context of you are.

1. What was your age at your last birthday?

[ ]

2. Which race/ethnicity best describes you? (Please choose only one.)

- ◯ American Indian or Alaskan Native
- ◯ Asian / Pacific Islander
- ◯ Black or African American
- ◯ Hispanic
- ◯ White / Caucasian
- ◯ Multiple ethnicity / Other (please specify)

[ ]

3. Are you of Spanish, Hispanic or Latino origin or descent?

- ◯ No, not Spanish/Hispanic/Latino
- ◯ Yes, Puerto Rican
- ◯ Yes, Mexican, Mexican American, Chicano
- ◯ Yes, Cuban
- ◯ Yes, other Spanish/Hispanic/Latino

4. What is the highest level of school you have completed or the highest degree you have received?

- Less than high school degree
- High school degree or equivalent (e.g., GED)
- Some college but no degree
- Associate degree
- Bachelor degree
- Graduate degree

Please rate the following things based on how helpful you think it is in helping people over 40 years old keep their brain healthy. After each thing, please tell us whether you're already doing it regularly (2 or 3 times per week).

* 5. **Regular aerobic exercise** (e.g., walking, swimming, bicycling, running).

Doesn't help	Helps a little	Helps a medium amount	Very helpful	Extremely helpful	Don't know	Prefer not to answer
○	○	○	○	○	○	○

* 6. I am doing this regularly

Yes	No	Prefer not to answer
○	○	○

* 7. **Strength training exercise** (e.g., weightlifting, using elastic resistance bands)

Doesn't help	Helps a little	Helps a medium amount	Very helpful	Extremely helpful	Don't know	Prefer not to answer
○	○	○	○	○	○	○

* 8. I am doing this regularly

Yes	No	Prefer not to answer
○	○	○

* 9. **Engaging in mentally stimulating activities** (e.g., reading, working puzzles, doing crafts)

Doesn't help	Helps a little	Helps a medium amount	Very helpful	Extremely helpful	Don't know	Prefer not to answer
○	○	○	○	○	○	○

* 10. I am doing this regularly

Yes	No	Prefer not to answer
○	○	○

* 11. **Using a computer several times per week** or more often to search for information, play games, or take care of finances.

Doesn't help	Helps a little	Helps a medium amount	Very helpful	Extremely helpful	Don't know	Prefer not to answer
◯	◯	◯	◯	◯	◯	◯

* 12. I am doing this regularly

Yes	No	Prefer not to answer
◯	◯	◯

* 13. **Learning a new language or a musical instrument**.

Doesn't help	Helps a little	Helps a medium amount	Very helpful	Extremely helpful	Don't know	Prefer not to answer
◯	◯	◯	◯	◯	◯	◯

* 14. I am doing this regularly

Yes	No	Prefer not to answer
◯	◯	◯

* 15. **Following a balanced diet rich in fruits and vegetables**, such as the Mediterranean or MIND diets

Doesn't help	Helps a little	Helps a medium amount	Very helpful	Extremely helpful	Don't know	Prefer not to answer
◯	◯	◯	◯	◯	◯	◯

* 16. I am doing this regularly

Yes	No	Prefer not to answer
◯	◯	◯

17. Regularly doing **computer-based cognitive training** (e.g., BrainHQ, Lumosity, Cognifit, Staying Sharp on the AARP website)

Doesn't help	Helps a little	Helps a medium amount	Very helpful	Extremely helpful	Don't know	Prefer not to answer
◯	◯	◯	◯	◯	◯	◯

18. I am doing this regularly

Yes	No	Prefer not to answer
◯	◯	◯

* 19. **Getting together once a month or more often with friends** or family to have dinner, play a game, or see a movie

Doesn't help	Helps a little	Helps a medium amount	Very helpful	Extremely helpful	Don't know	Prefer not to answer
◯	◯	◯	◯	◯	◯	◯

* 20. I am doing this regularly

Yes	No	Prefer not to answer
◯	◯	◯

* 21. **Engaging in creative activities** (e.g., painting, music, or writing).

Doesn't help	Helps a little	Helps a medium amount	Very helpful	Extremely helpful	Don't know	Prefer not to answer
◯	◯	◯	◯	◯	◯	◯

* 22. I am doing this regularly

Yes	No	Prefer not to answer
◯	◯	◯

* 23. **Practicing meditation or mindfulness.**

Doesn't help	Helps a little	Helps a medium amount	Very helpful	Extremely helpful	Don't know	Prefer not to answer
◯	◯	◯	◯	◯	◯	◯

* 24. I am already doing this regularly (at least 2-3 times a week).

Yes	No	Prefer not to answer
◯	◯	◯

* 25. **Getting regular, adequate sleep (at least 6-7 hours a night)**.

Doesn't help	Helps a little	Helps a medium amount	Very helpful	Extremely helpful	Don't know	Prefer not to answer
◯	◯	◯	◯	◯	◯	◯

* 26. I am already doing this regularly (at least 2-3 times a week).

Yes	No	Prefer not to answer
◯	◯	◯

* 27. **Reducing alcohol consumption**

Doesn't help	Helps a little	Helps a medium amount	Very helpful	Extremely helpful	Don't know	Prefer not to answer
◯	◯	◯	◯	◯	◯	◯

* 28. I have already done this.

Yes	No	I have already reduced my alcohol consumption or don't drink	Prefer not to answer
◯	◯	◯	◯

* 29. **Quitting smoking**

Doesn't help	Helps a little	Helps a medium amount	Very helpful	Extremely helpful	Don't know	Prefer not to answer
◯	◯	◯	◯	◯	◯	◯

* 30. I have already done this or I don't smoke tobacco.

Yes	No	I have already quit or don't smoke tobacco	Prefer not to answer
◯	◯	◯	◯

* 31. **Managing stress**

Doesn't help	Helps a little	Helps a medium amount	Very helpful	Extremely helpful	Don't know	Prefer not to answer
○	○	○	○	○	○	○

* 32. I am already doing this regularly (at least 2-3 times a week).

Yes	No	Prefer not to answer
○	○	○

* 33. **Getting regular medical check-ups** related to brain health, taking care of problems like diabetes, hypertension, high cholesterol, or thyroid problems

Doesn't help	Helps a little	Helps a medium amount	Very helpful	Extremely helpful	Don't know	Prefer not to answer
○	○	○	○	○	○	○

* 34. I am already doing this as my doctor says I should

Yes	No	Prefer not to answer
○	○	○

* 35. **Maintaining a healthy weight**

Doesn't help	Helps a little	Helps a medium amount	Very helpful	Extremely helpful	Don't know	Prefer not to answer
○	○	○	○	○	○	○

* 36. I am already doing this as my doctor says I should

Yes	No	Prefer not to answer
○	○	○

* 37. **Getting help for it if you’re depressed**

Doesn't help	Helps a little	Helps a medium amount	Very helpful	Extremely helpful	Don't know	Prefer not to answer
○	○	○	○	○	○	○

* 38. I am already doing this, or this isn't a problem for me

Yes	No	Prefer not to answer
◯	◯	◯

* 39. **Getting tested or using a hearing aid if you have trouble hearing**

Doesn't help	Helps a little	Helps a medium amount	Very helpful	Extremely helpful	Don't know	Prefer not to answer
◯	◯	◯	◯	◯	◯	◯

* 40. I am already doing this, or this isn't a problem for me

Yes	No	Prefer not to answer
◯	◯	◯

Brain Health Knowledge, Attitudes, and Preferences Questionnaire

* 41. **Taking brain health supplements** (e.g., vitamins, minerals).

Doesn't help	Helps a little	Helps a medium amount	Very helpful	Extremely helpful	Don't know	Prefer not to answer
○	○	○	○	○	○	○

* 42. I am already doing this regularly (at least 2-3 times a week).

Yes	No	Prefer not to answer
○	○	○

43. **Something else we haven’t asked you about (please tell us what it is).**

Brain Health Knowledge, Attitudes, and Preferences Questionnaire

How concerned are you?

This next section asks you about how concerned you are about your brain health and the kinds of things you might do to maintain or improve it.

* 44. **How important is maintaining good brain health to you?**

Not important	A little important	Moderately important	Very important	Extremely important	Prefer not to answer
◯	◯	◯	◯	◯	◯

* 45. **Have you taken any steps to improve your brain health in the past year? (Select all that apply)**

- ☐ Aerobic exercise (walking, running, bicycling, swimming)
- ☐ Resistance training (lifting weights, resistance bands, body weight exercises)
- ☐ Following a Mediterranean or MIND diet, high in fruits and vegetables with little meat or sweets
- ☐ Mentally stimulating activates (puzzles, reading, learning)
- ☐ Computer-based cognitive training (Cognifit, Lumosity, AARP Staying Sharp)
- ☐ Adequate sleep
- ☐ Stress management (meditation, yoga, progressive relaxation)
- ☐ Social engagement (seeing friends or families for group activities, volunteering)
- ☐ Other (please specify)
- ☐ None of the above

* 46. **How concerned are you about your brain health right now?**

Not concerned	A little concerned	Moderately concerned	Very concerned	Extremely concerned	Prefer not to answer
◯	◯	◯	◯	◯	◯

* 47. **I am currently doing something about my concerns**

Yes	No	Prefer not to answer
◯	◯	◯

* 48. **Have you ever experienced memory loss or cognitive difficulties?**

Yes	No	Prefer not to answer
◯	◯	◯

* 49. **Does anyone in your family have dementia or Alzheimer's disease?**

Yes	No	Prefer not to answer
○	○	○

* 50. **Would you consider changing your diet if you thought it could help your brain health?**

Yes	No	I'm already eating a healthy diet	Prefer not to answer
○	○	○	○

* 51. **Would you engage in regular exercise (at least two or three times a week) if you thought it could help you stay sharp or make it less likely that you would get dementia?**

Yes	No	I'm already exercising regularly	Prefer not to answer
○	○	○	○

* 52. **Would you regularly do other things, like meditation, cognitive training on a computer, or being more socially active (at least two or three times a week) if you thought it could help you stay sharp or make it less likely that you would get dementia?**

Yes	No	I'm already doing this regularly	Prefer not to answer
○	○	○	○

* 53. **If you were to focus on just one aspect of brain health, which would it be? (Please choose just one.)**

- ☐ Aerobic exercise (walking, running, bicycling, swimming)
- ☐ Resistance training (lifting weights, resistance bands, body weight exercises)
- ☐ Following a Mediterranean or MIND diet, high in fruits and vegetables with little meat or sweets
- ☐ Mentally stimulating activates (puzzles, reading, learning)
- ☐ Computer-based cognitive training (Cognifit, Lumosity, AARP Staying Sharp)
- ☐ Adequate sleep
- ☐ Stress management (meditation, yoga, progressive relaxation)
- ☐ Social engagement (seeing friends or families for group activities, volunteering)
- ☐ Other (please specify)
- ☐ None of the above

* 54. **If you believe that multiple factors contribute to brain health, which activities would you want to pursue in a plan of combined activities? (Choose all that you would do.)**

- ☐ Aerobic exercise (walking, running, bicycling, swimming)
- ☐ Resistance training (lifting weights, resistance bands, body weight exercises)
- ☐ Following a Mediterranean or MIND diet, high in fruits and vegetables with little meat or sweets
- ☐ Mentally stimulating activates (puzzles, reading, learning)
- ☐ Computer-based cognitive training (Cognifit, Lumosity, AARP Staying Sharp)
- ☐ Adequate sleep
- ☐ Stress management (meditation, yoga, progressive relaxation)
- ☐ Social engagement (seeing friends or families for group activities, volunteering)
- ☐ Other (please specify)
- ☐ None of the above

55. **Are there any other things you would do to improve your brain health? Please write it in below**

* 56. **How likely are you to talk to a healthcare professional about brain health?**

Not at all likely	A little likely	Moderately likely	Very likely	Extremely likely	Prefer not to answer
○	○	○	○	○	○

* 57. I have already talked to a doctor or other healthcare provider about my brain health.

Yes	No	Prefer not to answer
○	○	○

* 58. **What types of support or resources would be most helpful to you in maintaining brain health? (check all that you think would be good)**

- [ ] in-person educational workshops or classes
- [ ] Online education workshops or classes
- [ ] Working with a healthcare provider who can give me a test to find out how good my memory or thinking is
- [ ] Working with a healthcare provider to develop a brain health plan
- [ ] Working on my own to have a brain health plan
- [ ] Having an app on my phone that can test my memory and thinking
- [ ] Having an app on my phone that help me track what I do for my brain health, like exercise, diet, and mental activity.
- [ ] Listening to podcasts or videos online
- [ ] Working In a group with other people like me, face to face
- [ ] In a group with other people like me online
- [ ] In an online message board where I can exchange messages with other people like me
- [ ] Reading a regular newsletter from a professional who knows about brain health
- [ ] Texting to get quick answers to questions
- [ ] E-mail consultations with a healthcare provider.
- [ ] Other (please specify)

- [ ] None of the above

59. **Are there any other ways you might like to get help with brain health? Please write it in below**

60. **Do you have any other comments are suggestions about how we should ask these questions, or did we miss something important?**
